# Pedunculated Acral Keratotic Papule on an Ischemic Foot

**DOI:** 10.3390/dermatopathology13020018

**Published:** 2026-04-21

**Authors:** Janmesh D. Patel, Pooja A. Shet, Sara E. Dahle, Joshua M. Schulman, Marat D. Kazak

**Affiliations:** 1 Department of Dermatology, VA Northern California Health Care System, Sacramento VA Medical Center, 10535 Hospital Way, Mather, CA 95655, USA; pjanmesh1998@gmail.com (J.D.P.);; 2 Department of Dermatology, University of California Davis Medical Center, 4301 X St, Sacramento, CA 95817, USA; 3 Podiatry Section, Surgical Services, VA Northern California Health Care System, Sacramento VA Medical Center, 10535 Hospital Way, Mather, CA 95655, USA

**Keywords:** acquired digital fibrokeratoma, acral keratotic papule, hallux, chronic limb-threatening ischemia, endovascular revascularization

## Abstract

An 86-year-old man presented with a slowly enlarging, subject to chronic mechanical irritation, pedunculated keratotic papule on the plantar tip of the right hallux in the setting of severe peripheral arterial disease. Excision was deferred until after endovascular revascularization to reduce the risk of nonhealing and limb complications, after which the lesion was removed without wound sequelae. Histopathology demonstrated a conical papule with a central collagenous core and an overlying cap of compact hyperkeratosis. This case highlights key clinicopathologic features that distinguish acral keratotic tumors and underscores the importance of perfusion optimization when planning elective excision on an ischemic limb.

## Simple Summary

Acquired digital fibrokeratoma is a rare, noncancerous growth that usually appears as a small bump on a finger or toe. This report describes an unusual case on the great toe of an older man who also had severely poor blood flow to the foot. Because surgery on an ischemic toe can lead to poor healing, tissue loss, or infection, removal of the lesion was delayed until blood flow was improved with vascular treatment. The lesion was then removed without wound complications, and microscopic examination confirmed the diagnosis. This case highlights two important points for clinicians and pathologists: first, careful clinicopathologic review is needed to distinguish this lesion from other keratotic growths of the digits; second, blood flow should be considered when planning even minor procedures on the foot.

## 1. Case Presentation

**Figure 1:**
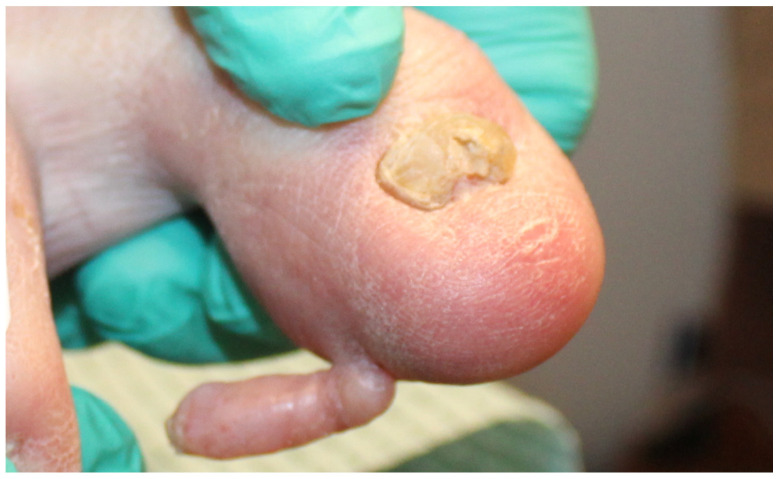
Preoperative clinical photograph of the right hallux showing a firm, pedunculated, hyperkeratotic papule with a collarette at the base.

**Figure 2:**
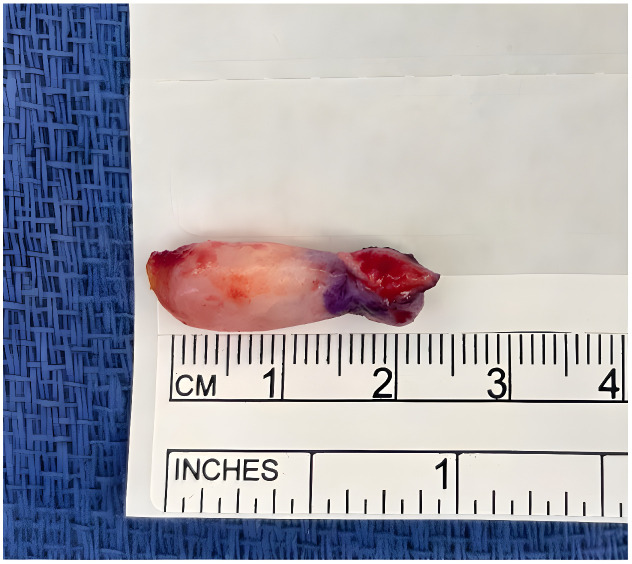
Gross photograph of the excised pedunculated lesion. Gross specimen demonstrates complete removal of the lesion.

**Figure 3:**
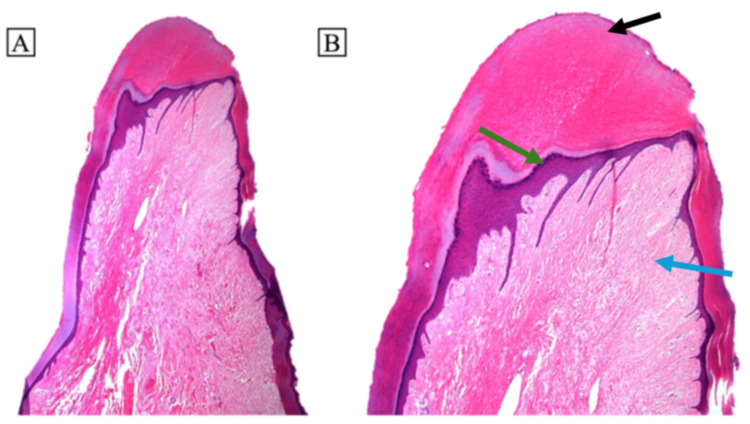
Histopathology of the distal tip of the lesion demonstrates a conical-shaped papule with a collagenous core (blue arrow), acanthotic epidermis (green arrow), and an overlying cap of compact hyperkeratosis (black arrow). (**A**) Hematoxylin and eosin, original magnification ×20; (**B**) hematoxylin and eosin, original magnification ×40.

An 86-year-old man presented with a slowly enlarging, subject to chronic mechanical irritation, pedunculated keratotic papule on the plantar tip of the right hallux for ~2 years. Examination revealed an elongated to cylindrical, flesh-colored, exophytic pedunculated papule measuring approximately 2 to 3 cm on the right great toe collarette (Figure 1). Objective perfusion testing before intervention demonstrated markedly impaired right-sided flow, with monophasic posterior tibial and dorsalis pedis waveforms, right great toe pressure of 41 mmHg, and a toe-brachial index of 0.27. The lesion progressively enlarged and became increasingly bothersome with shoe friction. The patient had been followed by podiatry, and conservative management was initially recommended (including accommodative footwear), but he requested removal because of discomfort and concern that the pedunculated lesion could catch during ambulation and cause him to trip or fall. Given that the patient had had severe peripheral arterial disease with nonpalpable pedal pulses, vascular clearance was pursued. Elective excision on an ischemic toe carries substantial risks of nonhealing, necrosis, infection, and even limb loss, and because the lesion lacked high-risk features for malignancy (no rapid growth, bleeding, or ulceration), the team deferred excision and pursued endovascular revascularization to restore inline flow and reduce perioperative wound risk [1]. Approximately six months after revascularization, with improved perfusion on examination, the lesion was excised (Figure 2). Grossly, the specimen consisted of an ellipse of skin measuring 0.9 × 0.5 × 0.2 cm with an attached oblong mass measuring 2.5 × 0.8 × 0.5 cm, confirming the complete excision of the clinically evident lesion. Histologically, the lesion showed a conical acral papule with compact hyperkeratosis, acanthotic epidermis, and a central fibrocollagenous core (Figure 3). In conjunction with the characteristic exophytic architecture and lack of features suggesting a myxoid or viral process, these findings were considered sufficient for diagnosis without ancillary immunohistochemical studies. The postoperative course was uneventful, and the excision site healed without dehiscence, infection, or ischemic necrosis. Re-evaluation of the vasculature 4 months post procedure revealed that the popliteal stent and anterior tibial artery remained patent without significant stenosis, and right great toe pressure improved to 61 mmHg.

### What Is the Diagnosis?

Which diagnosis best explains a pedunculated, keratotic acral papule with a collagenous core and vertically oriented collagen bundles on H&E?

- Acquired digital fibrokeratoma;
- Periungual fibrokeratoma;
- Superficial acral fibromyxoma;
- Accessory digit (rudimentary polydactyly);
- Verruca Vulgaris.

## 2. Diagnosis

- Acquired digital fibrokeratoma.

## 3. Discussion

ADF is an uncommon benign fibroepithelial tumor that typically presents as a solitary, dome- or rod-shaped, skin-colored to keratotic papule on fingers or toes. A collarette at the base is characteristic [2]. Histopathology shows compact hyperkeratosis and acanthosis overlying a central fibrocollagenous core that is composed of thick, vertically oriented collagen bundles with interspersed dilated capillaries, arranged along the longitudinal axis of the lesion, findings that are diagnostic in the proper clinical context [2,3,4]. The compact hyperkeratosis and associated epidermal hyperplasia/hypergranulosis in the distal tip are typical of ADF. In this plantar, chronically irritated location, the accentuation of hyperkeratosis at the apex may also reflect superimposed lichenification from repetitive pressure and friction. Complete excision at the base is curative in most cases, with low recurrence when the lesion’s stalk is fully removed [3].

The main differentials for a pedunculated acral keratotic tumor include the following:

Periungual fibrokeratoma (Koenen tumor) is an important clinical mimic because it also presents as a fibrous keratotic papule on a digit. However, it typically arises from the proximal or lateral nail fold, may produce a longitudinal groove or distortion of the nail plate, and is more closely tied to the nail apparatus than ADF [2,5]. In addition, the presence of multiple periungual fibrokeratomas should raise suspicion for tuberous sclerosis complex rather than a sporadic acquired lesion. In the present case, the lesion was located on the plantar-lateral hallux rather than in a periungual distribution, and there was no associated nail change, which made periungual fibrokeratoma less likely on anatomic and clinical grounds.

Superficial acral fibromyxoma (SAF) is the most important histopathologic pitfall in this setting because it also favors acral skin and may present as a slow-growing flesh-colored nodule. In the early clinicopathologic case series, these tumors occurred predominantly on fingers and toes, frequently involved the nail region, and were often present for years before excision [6]. Histologically, SAF is usually a dermal or subcutaneous proliferation of bland spindle and stellate cells arranged in loose storiform or fascicular patterns within a myxoid or fibromyxoid matrix, often with accentuated vasculature and increased mast cells. Immunohistochemically, it commonly shows CD34 positivity and frequent epithelial membrane antigen expression. By contrast, our lesion was distinctly exophytic, with a compact hyperkeratotic cap and a dense fibrocollagenous core oriented along the long axis of the lesion rather than a myxoid dermal proliferation, favoring ADF over SAF [6,7].

Accessory digit (rudimentary polydactyly) is another useful consideration because it can appear as a pedunculated fibrous papule, sometimes with a narrow stalk. The key distinguishing feature is clinical history, since accessory digits are congenital and typically recognized at birth or in early life, whereas ADF is acquired later in adulthood. Histologically, accessory digits may contain a neurovascular core and occasionally cartilage, findings not expected in ADF. In this case, the lesion was late-onset, progressively enlarging, and histologically lacked congenital appendage-type elements, arguing against rudimentary polydactyly [5].

Verruca vulgaris typically shows punctate “black dots” (thrombosed capillaries) that become evident after paring surface keratin. Histologically, it features circumscribed papillomatosis with compact hyperkeratosis and hypergranulosis, tightly packed capillaries in elongated dermal papillae, and koilocytosis/viral inclusions [2]. Long-standing (inveterate) plantar warts may become markedly hyperkeratotic and mimic a fibrous acral papule clinically, but they retain viral cytopathic change (koilocytosis) and papillomatosis rather than a central fibrocollagenous core with vertically oriented collagen bundles [2].

Sclerotic fibroma (storiform collagenoma) is less likely clinically but remains a useful microscopic consideration because it is also a fibrous lesion with dense collagenization. Classically, it is a sharply circumscribed, hypocellular dermal nodule composed of hyalinized collagen bundles arranged in a whorled or storiform pattern with characteristic clefting. That architecture differs from the longitudinally oriented fibrocollagenous core and exophytic cone-like configuration seen in ADF. In addition, sclerotic fibroma more often presents as a firm intradermal papule than as a pedunculated collarette-based acral projection [8].

Infantile digital fibroma (inclusion body fibromatosis) occurs almost exclusively in infants and young children, typically as firm nodules on fingers or toes, and is characterized histologically by myofibroblastic proliferation containing distinctive eosinophilic intracytoplasmic inclusion bodies. The patient’s advanced age and the absence of inclusion bodies make this diagnosis implausible in the present case. Its value in the differential is mainly to emphasize that not all digital fibrous tumors with dense stroma are variants of acquired fibrokeratoma [9].

Taken together, the lesion’s plantar-lateral location, pedunculated exophytic contour, collarette, and classic fibrocollagenous core with vertical collagen bundles most strongly supported ADF over these alternative acral fibrous and verrucous processes.

In this patient, the classic clinicopathologic presentation supported ADF, but management still required balancing two competing risks: proceeding with excision on an ischemic toe, with risks of nonhealing, infection, gangrene, and amputation, versus leaving in place a progressively enlarging, chronically traumatized lesion on a pressure-bearing site. This balance is particularly relevant in podiatric and dermatologic practice because lesions that are benign histologically may still justify removal when they create pain, recurrent friction, gait disturbance, or fall risk. Contemporary chronic limb-threatening ischemia guidelines support optimizing perfusion before nonurgent procedures to improve wound healing, while recognizing that lesions with rapid growth, ulceration, bleeding, marked induration, or other features concerning malignancy may require earlier biopsy or excision despite vascular risk. In our patient, the lesion enlarged slowly over approximately two years, remained flesh-colored, and lacked overtly aggressive features, which lowered oncologic urgency and favored revascularization first. This approach was supported by objective hemodynamic impairment before intervention and by documented postoperative improvement in toe pressure and patency of the treated vessels. The subsequent uncomplicated wound healing suggests that perfusion optimization can be clinically meaningful even for minor toe procedures when baseline ischemia is severe.

## Data Availability

No new datasets were generated for this study. All relevant data supporting the findings of this case report are contained within the article. Additional de-identified clinical details are not publicly available due to patient privacy considerations.
